# Improvement in the epigenetic modification and development competence in PCOS mice oocytes by hydro-alcoholic extract of *Nigella sativa* during in-vitro maturation: An experimental study

**DOI:** 10.18502/ijrm.v13i9.7668

**Published:** 2020-09-20

**Authors:** Fatemeh Eini, Khojasteh Joharchi, Maryam Azizi Kutenaei, Pegah Mousavi

**Affiliations:** ^1^Fertility and Infertility Research Center, Hormozgan University of Medical Sciences, Bandar Abbas, Iran.; ^2^Department of Pharmacology, School of Medicine, Shahid Beheshti University of Medical Sciences, Tehran, Iran.; ^3^Department of Medical Genetics, Faculty of Medicine, Hormozgan University of Medical Sciences, Bandar Abbas, Iran.

**Keywords:** Epigenetic modification, In-vitro maturation, Nigella sativa, Oxidative stress, Polycystic ovary syndrome

## Abstract

**Background:**

Nigella Sativa (NS) and its active component, thymoquinone, have beneficial protective effects on experimental animal models of polycystic ovary syndrome (PCOS) and different human diseases.

**Objective:**

The present study aimed to investigate the effects of NS hydro-alcoholic extract (NSE) on the oocyte quality of PCOS mice during in vitro maturation.

**Materials and Methods:**

For induction of PCOS, 40 prepubertal 21-days old female B6D2F1 mice (18-22 g body weight) received subcutaneous dehydroepiandrosterone daily. After validation of the model, germinal vesicle-stage oocytes of superovulated mice were collected and placed in the culture medium containing different concentrations (0, 1, 50, and 100 μg/ml) of NSE. For the measurement of developmental competency, some mature oocytes were fertilized with epididymal spermatozoa. Other mature oocytes were assessed for oxidative stress. Also, some mRNA expression levels involved in oocyte maturation and epigenetic modification were evaluated.

**Results:**

The 50 μg/ml NSE treated group showed significantly higher r ates o f maturation, f ertilization, and blastocyst formation in comparison with both control and PCOS groups. A high level of glutathione concentration and glutathione peroxidase mRNA expression, besides a low level of reactive oxygen species content all, were observed in oocytes treated with 50 μg/ml NSE, indicating the modification of oxidative statue. Furthermore, the oocytes in the 50 μg/ml-treated group showed an upregulation of mRNA expression in epigenetic-related genes (*Dnmt1* and *Hdac1*) and maternally derived genes (*Mapk* and *Cdk1*), correspondingly downregulation of cyclooxygenase2 mRNA expression, in comparison to other groups.

**Conclusion:**

The results of this study indicated that 50 μg/ml NSE improves oocyte maturation, oxidative statues and epigenetic modifications. These may be the all reasons for the developmental competency in the control and PCOS mice oocytes.

## 1. Introduction

In vitro maturation (IVM) of oocytes has been introduced as an alternative treatment to traditional stimulated in vitro fertilization (IVF) technique, as IVM could be safely used by patients who are at a high risk of ovarian hyperstimulation, especially polycystic ovary syndrome (PCOS) (1). Although many newborns have been registered after human IVM treatment, in vitro condition can be improved by natural supplements (2). Adding typical supplements such as cysteine, homocysteine, melatonin, vitamin E, vitamin C, etc. can upgrade basal clinical IVM media (3-5). The beneficial protective activities of these additives are attributed to the potent antioxidant property during IVM. *Nigella sativa* (NS) and its potent constituent, thymoquinone (TQ), is widely known as a medicinal plant for improving general health due to the various pharmacological properties and therapeutic potentials (6).

A basic animal study suggested that NS with adequate concentration has many therapeutic effects such as anti-inflammatory (7) and antioxidant (8) properties. Furthermore, TQ induces some endogenous antioxidants such as superoxide dismutase, catalase, glutathione S-transferases (GST), glutathione peroxidase (GPX), and glutathione (GSH), all modulating oxidative stress in a suboptimal state like organ dysfunction (6). However, it should be noted that the beneficial effects of NS may not be limited only due to the presence of TQ, because other components in the extract such as GSH and vitamins may also influence oocyte maturation (9). Epigenetic modification showed to be affected by the culture medium and its ingredients (10). Generally, epigenetic modification is modulated by enzymes such as DNA methyltransferase and histone deacetylase, the key regulators during oocyte maturation (11, 12). Chromosomal condensation, appropriate stored mRNA levels, and synchronized nuclear and cytoplasmic maturation are three crucial factors for the developmental competency in oocyte (13, 14). At present, there is a gap of knowledge about how epigenetic modification aspects could be changed by antioxidant supplements. A recent study reported that hyperandrogenism and reactive oxygen species (ROS) production impaired chromosome condensation in PCOS mice model (15).

Therefore, in this study, NS extract was taken into account due to some competent activities which directly modulated epigenetic-related enzymes during IVM. Besides, nuclear and cytoplasmic maturation, ROS and GSH contents, oxidative stress levels, and expression of epigenetic-related genes in oocytes were investigated for both control and PCOS mice models. The effects of the different concentrations of NS hydro-alcoholic extract (NSE) were investigated on oocyte quality along with the potential mechanisms involved in this development.

## 2. Materials and Methods

In this experimental study, all chemicals were purchased from Sigma-Aldrich Chemical Co. (St. Louis, MO, USA) (exceptions included).

### Preparation of alcoholic NS extraction 

The seeds of NS were purchased from a local herb market and the taxonomic identification of the seeds was confirmed by a senior plant taxonomist while giving the herbarium number of PMP-1735. The extract was prepared according to the Word Health Organization (WHO) protocol CG-04 (16). The dried seeds (100 gr) were powdered and then applied to Soxhlet for the extraction with 50% ethanol. The resultant alcoholic extract was filtered using a filter paper (Whatman No. 3) and then evaporated at 40°C to dry in vacuum. The extract was stored at -20°C until use (yield 12%). A stock solution of NSE was prepared in dimethyl sulfoxide (DMSO), and suitable working concentrations were prepared from the stock using a complete medium. The stock solution of NS was prepared as 100 mg/ml in DMSO, and was diluted in IVM medium to reach the final concentrations of 0, 1, 50, and 100 µg/ml. In the control group, oocytes were only cultured in IVM medium containing 0.1% (v/v) of DMSO (final concentration of DMSO ≤ 0.1%). No difference was observed between the presence or absence of DMSO, indicating that the DMSO at the tested concentrations did not influence the results.

### Induction and validation of PCOS mice model

In this experimental study, 80 prepubertal 21-day old female B6D2F1 (18-22 g body weight) mice were randomly divided into two groups, treatment and control (n = 40/ each). They were housed in an animal room with an ambient temperature of 20-24°C and a 12 hr light/dark cycle. All mice had free access to food and water. They received a daily subcutaneous shot of either a mixture of 100 µl solution of dehydroepiandrosterone (DHEA) 60 mg/kg body weight dissolved in a 9/1 mixture of sesame oil/95% ethanol (treatment) or 100 µl sesame oil/95% ethanol (control) for 20 consecutive days. Model validity was described and confirmed by acyclicity, ovarian histopathology and hormonal abnormality in our recent study (15, 17). During oocyte quality assessment, 2 to 3 mice per group were used in each experiment except for 3 or 5 per group used in IVM and IVF.

### Collection of mice oocytes

After PCOS confirmation, the mice in both groups, 6-8 wks of age, were superovulated with intraperitoneal injection of 10 IU pregnant mare's serum gonadotropin and sacrificed 46-48 hr later. Both the ovaries were excised and washed twice in 37°C sterile physiological saline (containing 100 IU/L penicillin and 50 mg/L streptomycin). Cumulus oocyte complexes (COCs) were physically retrieved fromantral follicles (2-8 mm in diameter) in HEPES-buffered tissue culture medium using a pair of 27-G needles under a stereomicroscope (Nikon, SMZ645, Tokyo, Japan). The buffer was improved with 5% (v/v) heat-inactivated fetal bovine serum (FBS; GIBCO BRL Invitrogen, USA). After washing 3-times with tissue culture medium (TCM-199), only the COCs with at least three layers of compact cumulus cells and uniform ooplasm were taken as normal for use in all experiments. Approximately 20-30 COCs per animal were isolated from the mice (17).

### In vitro maturation

Maturation medium contained of TCM-199, supplemented with 10 μg/mL follicle-stimulating hormone (FSH), 10 μg/mL luteinizing hormone (LH), 10% (v/v) fetal bovine serum (FBS, 24.2 mg/l sodium pyruvate, and 1 μg/ml 17-β estradiol at 37°C and 5% CO${}_{2}$. Then, immediately, NSE from prepared stock was added to the maturation medium for preparation of different IVM groups (0, 1, 50, and 100 µg/ml). After that, a total number of 10-15 normal COCs were cultured in 50 µL microdrop of 4 mentioned concentrations. The oocytes were then separated from cumulus cells by mechanical pipetting after 22-24 hrs. The first polar body extrusion was considered as the criteria for the metaphase II (MII)-stage oocyte rate. Moreover, oocytes were selected according to normal morphological criteria with a round, clear zona pellucida, a small perivitelline space, and a pale moderately granular cytoplasm that did not contain inclusions (18). Only normal oocytes were used in following tests.

### In vitro fertilization and embryo culture

After oocyte maturation, IVF was performed with normal spermatozoa retrieved from the cauda-epididymis and vas deferens of 10-12-wk-old B6D2F1 male mice in different IVM groups. The washed sperm were capacitated in enhanced ham's-F10 with 5 mg/ml BSA. Then, Groups of 20 MII oocytes were placed to 120 μl fertilization droplets (potassium simplex optimized medium, KSOM, with 15 mg/ml BSA) with capacitated spermatozoa at the concentration of about 3 × 10${}^{6}$/ml. Then, dishes were incubated for 6 hrs. at 37°C in a humidified 5% CO${}_{2}$ atmosphere. Fertilization was evaluated by the observation of two pronuclei (2PN). Finally, the culture was done in 30 μl of culture droplets (KSOM supplemented with 4 mg/ml BSA) (one embryo per 2 µl) under mineral oil. The day of insemination was taken as day 0, and the cleavage rate was evaluated 24 hrs. later. The blastocyst rate was documented at the end of day 5 (17).

### Measurement of intracellular ROS and GSH levels

In order to detection of intracellular ROS and GSH levels, the 2',7'-dichlorodihydrofluorescein diacetate (H${}_{2}$DCFDA; Cell Tracker Green; Molecular Probes, Invitrogen, USA) and 4-chloromethyl-6, 8-difluoro-7-hydroxycoumarin (CMF2HC; Cell Tracker Blue; Molecular Probes, Invitrogen, USA) were used. For this, the oocytes were incubated for 30 mins. At 37°C in 10 µM droplet of each marker as described previously (19). The incubated oocytes were analyzed for florescence intensity using fluorescence microscope (Labomed Lx 400; Labo America) with ultraviolet filters (460 nm for intracytoplasmic ROS, 370 nm for GSH). The details were described in our recent study (17).

### RNA isolation and quantitative real-time polymerase chain reaction

After denuding and washing of mature oocytes, 10-15 oocytes were accumulated four times, and total RNA was isolated from these accumulations. The RNeasy Micro kits (Qiagen Inc., Valencia, CA, USA) and transcript nova kit (Qiagen Inc., Valencia, CA, USA) were used for RNA isolation and reverse-transcription into cDNA as a described previously (20). The reverse-transcribed yields of two subunits of maturation-promoting factor (*MPF: Cdk1*, *cyclin B)*, mitogen-activated protein kinase (*Mapk), *Cyclooxygenase (*Cox2*), Glutathione peroxidase (Gpx1), DNA methyltransferase-1 (*Dnmt1)* and histone deacethylase-1 (*Hdac1)* were amplified by real-time polymerase chain reaction (PCR) with SYBR Green (Takara, Japan) on an ABI real-time PCR system (Applied Biosystems, ABI, Foster City, CA, USA), according to the manufacturer's instructions. Finally, all data were analyzed by the standard formula, while glyceraldehyde-3-phosphate dehydrogenase (*Gapdh*) was used as an internal reference gene. The details of real-time PCR reaction was described in our previous article (17). Table I lists the primer sequences.

**Table 1:** Primer sequences


**Gene**	**Primer sequences (5'-3')**	**Accession number**	**length**	**TM (°C)**
*Dnmt1*	F:CCTAGTTCCGTGGCTACGAGGAGAA R:TCTCTCTCCTCTGCAGCCGACTCA	NM_010066	137	58
*Hdac1*	F:CTGAATACAGCAAGCAGATGCAGAG R:TCCCGTGGACAACTGACAGAAC	NM_008228	92	57
*Cox2*	F:GAAGTCTTTGGTCTGGTGCCT R:GCTCCTGCTTGAGTATGTCG	NM_011198	91	60
*Gpx1*	F:CGC TTT CGT ACC ATC GAC ATC R:GGG CCG CCT TAG GAG TTG	NM_001329527	77	59
*Cdk1*	F:TTGGAGAAGGTACTTACGGTGTGGTG R:CCAGGAGGGATGGAGTCCAGGT	NM_007659	89	58
*Cyclin B*	F:CTGACCCAAACCTCTGTAGTG R:CCTGTATTAGCCAGTCAATGAGG	NM_172301	150	61
*Mapk*	F:TTTGCATAGGGAGGTCCAAG R:GGTGCCATCATCAACATCTG	NM_0119449	150	60
*Gapdh*	F:TGACGTGCCGCCTGGAGAAA R:AGTGTAGCCCAAGATGCCCTTCAG	NM_008084	98	58
*Dnmt1*: DNA methyltransferase1, *Hdac1*: Histone deacethylase1, *Cox2*: Cyclooxygenase, *Gpx1*: Glutathione peroxidase, *Cdk1*: Cyclin-dependent kinase 1, *Mapk*: Mitogen-activated protein kinase, *Gapdh*: Glyceraldehyde-3-phosphate dehydrogenase				

### Ethical consideration 

All study procedures were conducted regarding guidelines of the International Association (http://www.iasp-pain.org) to reduce animal's pain (21), and were approved by the Internal Deputy for Animal Study, based on local Institutional Animal Welfare law, which is recommended by an independent research ethics committee in Shahid Beheshti University of Medical Sciences with the ethical committee code: IR.SBMU.RAM.REC.1394.523 (17).

### Statistical analysis

All experiments were run in triplicate, and the data were presented as mean ± SEM. Student's *t* test was used to analyze all measurements between PCOS and control group. A one-way analysis of variance (ANOVA), followed by Duncan's multiple comparisons tests were performed using Prism v5 (Graphpad Software, Inc., San Diego, CA) for all measurements between four concentrations of NSE in both control and PCOS groups. Two-tailed p < 0.05 was taken as the significance level. The fluorescence intensities of oocytes were analyzed using Image J, version 1.40 (National Institutes of Health) and were normalized against values in the control group.

## 3. Results

### Effect of NSE on oocyte maturation

Table II and III summarizes the results of oocyte maturation and abnormal morphologh in both control and PCOS groups with different concentrations of NSE during IVM. They show that MII rate was significantly higherand the abnormality rate was lower in the control group than in the PCOS model with 0 and 1 µg/ml NSE concentrations respectively (Table II). The percentage of MII oocytes in the PCOS oocytes was significantly higher in 1, 50, and 100 µg/ml of NSE than the control. However, only supplementation with 50 µg/ml NSE had a strong effect on the reduction of abnormality rate in PCOS-treated oocytes as compared to the control. In the control group, the MII rate was significantly higher in 1 and 50 µg/ml of NSE concentrations when compared to the control. Besides, the reduction of abnormality was significantly lower in 1 and 50 µg/ml NSE-treated oocytes as compared to the control. Finally, a comparison of abnormality rate for different concentrations of NSE showed that lower and higher-value concentrations cannot reduce the abnormality rate (Table III).

### Effect of NSE on embryonic development

Table IV and V summarizes the results of embryonic development following the maturation of oocytes, via IVM. Fertilization, 2-cell, 8-cell, and blastocyst rates were significantly higher in the control than in the PCOS group of untreated oocytes. In 1 µg/ml NSE group only fertilization rate was higher in the control than in the PCOS group (Table IV). In the control mice, the fertilization rate was not different across different concenterations of NSE; however, the 2-cell, 8-cell, and blastulation rates were significantly higher in 50 µg/ml NSE-treated oocytes in comparison with the untreated. Moreover, fertilization, 2-cell, 8-cell, and blastulation rates were significantly higher in the 50 µg/ml NSE-treated PCOS mice oocytes than the untreated (Table V).

### Antioxidant effect of NSE on ooplasmic GSH and ROS levels and *Gpx1* gene expression 

**Figure 1:**
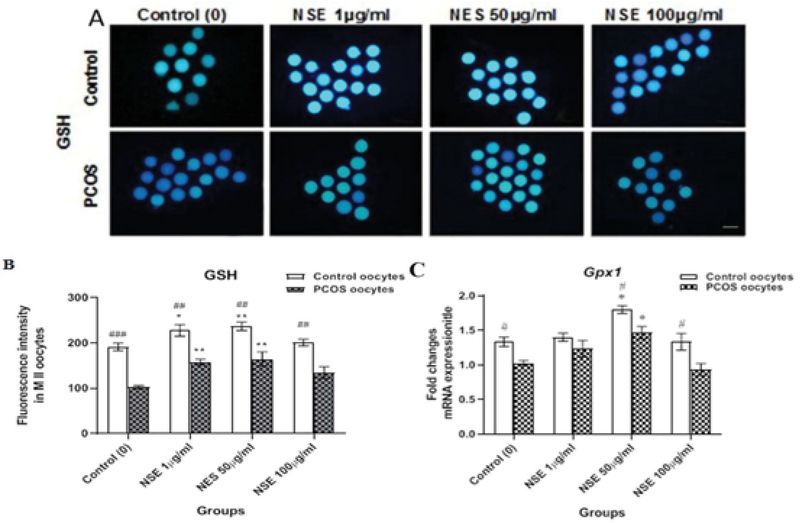
The GSH level of in vitro-matured control and PCOS mice oocytes according to the different concentrations of NSE in the maturation medium. (A) Representative images of intracellular GSH levels in the control and PCOS mice. (B) Quantification data. (C) The relative mRNA expressions of *Gpx1* gene of in vitro-matured control and PCOS mice oocytes according to the different concentrations of NSE in the maturation medium. Scale bars = 100 μm. Data are expressed as Mean ± SEM. *P < 0.05 and **P < 0.01 vs. control (0μg/ml) by one-way ANOVA. ${}^{\#}$P < 0.05, ${}^{\#\#}$P < 0.01, ${}^{\#\#\#}$P < 0.001, between two groups (Control vs. PCOS) by student's *t* test.

**Figure 2:**
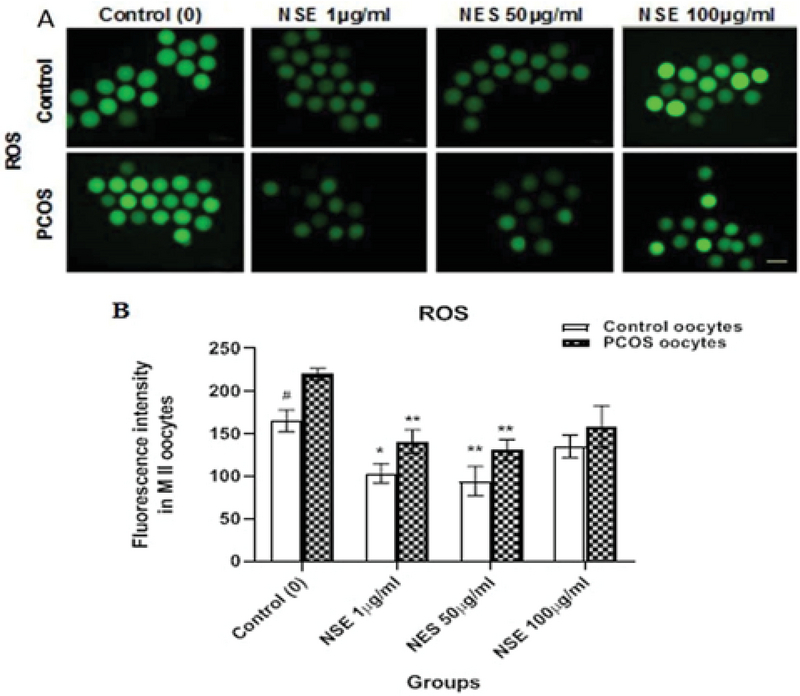
The ROS level of in vitro-matured control and PCOS mice oocytes according to the different concentrations of NSE in the maturation medium. (A) Representative images of intracellular ROS level in the control and PCOS mice. (B) Quantification data. Scale bars = 100 μm. Data are expressed as Mean ± SEM. *P < 0.05 and **P < 0.01 vs. control (0 μg/ml) by one-way ANOVA. ${}^{\#}$P < 0.05, between two groups (Control vs. PCOS) by student's *t* test.

The CellTracker${}^{\mathrm{TM}}$ Blue and Green dyes were used to detect intracytoplasmic GSH (Figure 1) as blue fluorescence and ROS (Figure 2) as green fluorescence, respectively. In these two figures, the enhanced fluorescent intensity of the oocytes is representative of more GSH and ROS levels. In this regard, our results showed that the GSH and *Gpx1 *mRNA expression levels were significantly higher in the control than the PCOS. The details are shown in Figure 1. Moreover, both the control and PCOS oocytes showed an increase in intracellular GSH level in the 1 (227.9 ± 12.64 vs 191.3 ± 8.38, p = 0.017 and 158 ± 6.32 vs 102.83 ± 3.14, p = 0.0083) and 50 (236.9 ± 12.64 vs 191.3 ± 8.38, p = 0.0057 and 164.67 ± 15.8 vs 102.83 ± 3.14, p = 0.0023) µg/ml NSE-treated oocytes as compared to the untreated oocytes, respectively (Figure 1). However, the intracellular ROS level was significantly lower in the control group than the PCOS. Furthermore, the intracellular ROS levels were significantly lower in the control and PCOS oocytes treated with 1 (103.9 ± 11.46 vs 165.4 ± 12.86, p = 0.023 and 141 ± 13.85 vs 220.21 ± 6.97, p = 0.0067) and 50 (94.7 ± 17.0 vs 165.4 ± 12.86, p = 0.0081; 131 ± 12.19 vs 220.21 ± 6.97, p = 0.0035) µg/ml NSE as compared to untreated oocytes, respectively (Figure 2). In addition, the *Gpx1 *expression was significantly higher in 50 µg/ml NSE-treated group versus untreated oocytes in both control (1.8 ± 0.06 vs 1.33 ± 0.06, p = 0.043) and PCOS (1.47 ± 0.08 vs 1.03 ± 0.03, p = 0.015) groups (Figure 1).

### Effect of NSE on *MPF (Cdk1 & Cyclin B)* and *MAPK* genes expression

**Figure 3:**
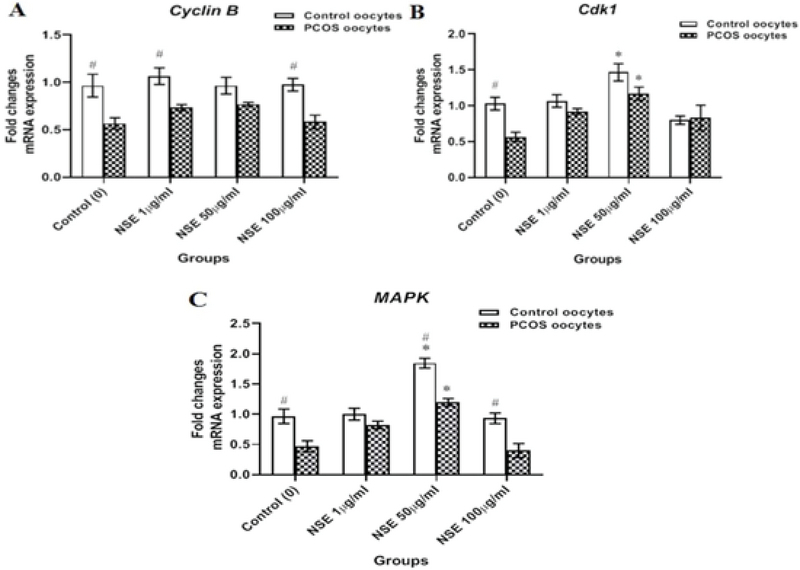
The relative mRNA expressions of nuclear maturation-related genes of in vitro-matured control and PCOS mice oocytes according to the different concentrations of NSE in the maturation medium. (A) *Cyclin B* mRNA expression. (B) *Cdk1* mRNA expression. (C) *Mapk* mRNA expression. Data are expressed as Mean ± SEM. *P < 0.05 and **P < 0.01 versus control (0 μg/ml) by one-way ANOVA. ${}^{\#}$P < 0.05, between two groups (Control vs. PCOS) by student's *t* test.

As an assessment of oocyte nuclear maturation, three nuclear maturation-related genes were evaluated in real-time PCR. In the control group, *Cyclin B *(0.96 ± 0.09 vs 0.56 ± 0.06, p = 0.022), *Cdk1* (1.03 ± 0.12 vs 0.56 ± 0.09, p = 0.035), and *Mapk* (0.96 ± 0.18 vs 0.47 ± 0.13, p = 0.041) mRNA levels were significantly higher than PCOS. The details are shown in Figure 3. However, the *Cdk1* mRNA expression was significantly higher in the 50 µg/ml NSE-treated oocytes than that the untreated in both control (1.47 ± 0.11 vs 1.03 ± 0.05, p = 0.037) and PCOS (1.17 ± 0.23 vs 0.56 ± 0.09, p = 0.012). Although, treatment with NSE had no effect on the gene expression of *Cyclin B*., the *Mapk* mRNA expression in both control (1.85 ± 0.06 vs 0.96 ± 0.18, p = 0.025) and PCOS (1.2 ± 0.09 vs 0.47 ± 0.13, p = 0.0067) mice oocytes was significantly higher in 50 µg/ml NSE-treated oocytes than in the untreated (Figure 3).

### Effect of NSE on *Cox2* genes expression 

**Figure 4:**
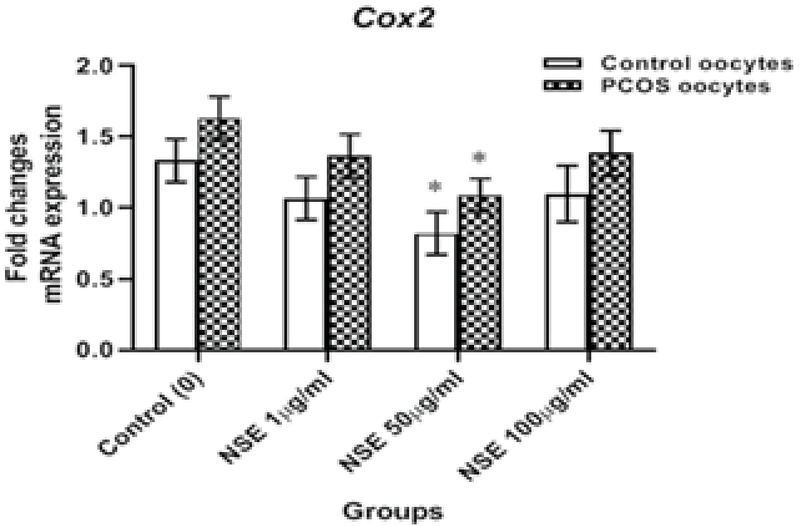
The Relative mRNA expressions of cyclooxygenase (*Cox2*) gene of in vitro-matured control and PCOS mice oocytes according to the different concentrations of *Nigella sativa* hydro-alcoholic extract (NSE) in the maturation medium. *P < 0.05 vs. control (0 μg/ml) by one-way ANOVA.

In order to assess the inhibitory effect of NSE on the apoptotic pathway, the *Cox2 *gene expression was evaluated in mature oocytes. *Cox2* mRNA expression was significantly decreased in the 50 µg/ml NSE-treated oocytes as compared to the untreated in both control (0.82 ± 0.08 vs 1.33 ± 0.08, p = 0.047) and PCOS (1.09 ± 0.06 vs 1.63 ± 0.08, p = 0.035) groups. Moreover, the results showed that there was no significant difference in *Cox2* expression between the PCOS and control mice in all groups of NSE concentrations (Figure 4).

### Effect of NSE on the expression of epigenetic-related gene *(Hdac1 and Dntm1)*

**Figure 5:**
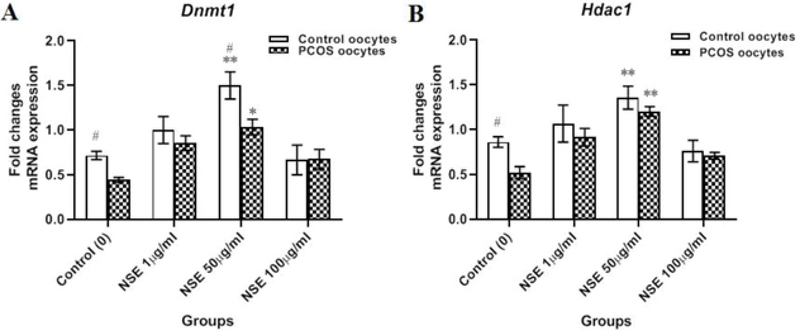
(A) The relative mRNA expression of DNA methyltransferase1 (*Dnmt1*) gene of in vitro-matured control and PCOS mice oocytes according to the different concentrations of *Nigella sativa* hydro-alcoholic extract (NSE) in the maturation medium. (B) The relative mRNA expression of histone deacetylase (*Hdac1) *gene of in vitro-matured control and PCOS mice oocytes according to the different concentrations of NSE in the maturation medium. Data are expressed as Mean ± SEM. *P < 0.05 and **P < 0.01 vs. control (0 μg/ml) by one-way ANOVA. ${}^{\#}$P < 0.05, between two groups (Control vs. PCOS) by student's *t* test.

Epigenetic defects particularly reduce DNA methylation and interfere with the transcription of certain enzymes in oocytes. The expression of enzymes may also be affected by ROS production. Therefore, our results showed that the *Dnmt1* and *Hdac1* mRNA levels were significantly higher in the control than the PCOS group. The details are shown in Figure 5. Moreover, during IVM, treatment with 50 µg/ml NSE resulted in higher gene expression of *Dnmt1 *(1.4 ± 0.17 vs 0.56 ± 0.09, p = 0.0071), (1.24 ± 0.03 vs 0.54 ±0.03, p = 0.014) and *Hdac1* (1.1 ± 0.12 vs 0.52 ±0.09, p = 0.0097), (1.06 ± 0.12 vs 0.52 ±0.04, p = 0.0065) levels in comparison to untreated oocytes in both control and PCOS mice, respectively (Figure 5).

**Table 2:** The comparision of different concentrations of NSE on oocyte maturation and abnormal morphology between the control and PCOS mice oocytes during in vitro maturation


**Groups**	**MII (%)**			**Abnormal morphology (%)**		
	**Control**	**PCOS**	**P-value**	**Control**	**PCOS**	**P-value**
**Control (0)**	84.93 ± 1.10, n =146	71.38 ± 3.61, n =121	0.025	4.51 ± 0.18	10.57 ± 1.06	0.012
**NES 1 μg/ml**	93.52 ± 1.98, n =167	83.00 ± 2.45, n =105	0.043	3.10 ± 0.71	6.87 ± 0.64	0.037
**NES 50 μg/ml**	93.63 ± 2.29, n =130	86.75 ± 2.10, n =123	0.134	2.98 ± 0.60	3.96 ± 0.80	0.971
**NES 100 μg/ml**	87.08 ± 1.86, n = 135	83. 86 ± 2.54, n =111	0.865	4.87 ± 0.35	8.72 ± 0.36	0.046
MII %: The percentage of mature oocytes; Abnormal morphology %: The percentage of oocyte with abnormal morphology after maturation; NES: Nigella sativa extract; PCOS: Polycystic ovary syndrome. N: number of oocytes. Values are expressed as Mean ± SEM; P-value are presented between two groups (Control versus PCOS) by student's *t* test						

**Table 3:** The effect of different concentrations of NSE on oocyte maturation and abnormal morphology in the control and PCOS mice oocytes during in vitro maturation


**Groups**	**MII (%)**		**Abnormal morphology (%)**	
	**Control mice**	**PCOS mice**	**Control mice**	**PCOS mice**
**Control (0)**	84.93 ± 1.10, n = 146	71.38 ± 3.61, n = 121	4.51 ± 0.18	10.57 ± 1.06
**NES 1 μg/ml**	93.52 ± 1.98, n = 167 (p = 0.031)	83.00 ± 2.45, n = 105 (p = 0.042)	3.10 ± 0.71 (p = 0.027)	6.87 ± 0.64 (p = 0.145)
**NES 50 μg/ml**	93.63 ± 2.29, n = 130 (p = 0.027)	86.75 ± 2.10, n = 123 (p = 0.002)	2.98 ± 0.60 (p = 0.018)	3.96 ± 0.80 (p = 0.0004)
**NES 100 μg/ml**	87.08 ± 1.86, n = 135 (p = 0.556)	83. 86 ± 2.54, n = 111 (p = 0.032)	4.87 ± 0.35 (p = 0.728)	8.72 ± 0.36 (p = 0.723)
MII %: The percentage of mature oocytes; Abnormal morphology %: The percentage of oocyte with abnormal morphology after maturation; NES: Nigella sativa extract; PCOS: Polycystic ovary syndrome. N: number of oocytes. Values are expressed as Mean ± SEM. P: P-value between each concentration group of NSE and control (0 μg/ml of NSE) by one-way ANOVA				

**Table 4:** The comparision of different concentrations of NSE on embryonic development between the control and PCOS mice oocytes during in vitro maturation


**Groups**	**Fertilization (%)**		**2-cell (%)**		**8-cell (%)**		**Blastocyst (%)**	
	**Control**	**PCOS**	**Control**	**PCOS**	**Control**	**PCOS**	**Control**	**PCOS**
**Control (0)**	78.50 ± 2.45 (p = 0.038)	68.50 ± 2.34	66.83 ± 2.84 (p = 0.025)	55.16 ± 3.57	49.00 ± 4.11 (p = 0.015)	40.67 ± 4.12	42.50 ± 3.16${}^{\#}$ (p = 0.045)	30.83 ± 2.54
**NES 1 μg/ml**	87.50 ± 2.67 (p = 0.042)	75.16 ± 2.18	73.17 ± 3.52 (p = 0.831)	69.83 ± 2.77	51.66 ± 3.47 (p = 423)	45.00 ± 4.10	46.33 ± 2.86 (p = 128)	37.00 ± 3.52
**NES 50 μg/ml**	88.83 ± 1.85 (p = 0.242)	82.17 ± 1.9	81.50 ± 2.86 (p = 0.541)	77.50 ± 2.41	65.00 ± 3.01 (p = 0.971)	63.33 ± 3.86	57.83 ± 2.70 (p = 0.089)	50.33 ± 2.94
**NES 100 μg/ml**	76.17 ± 3.10 (p = 0.098)	71.17 ± 2.98	69.00 ± 3.52 (p = 0.086)	59.50 ± 3.22	48.00 ± 3.61 (p = 0.121)	44.67 ± 3.45	42.50 ± 3.56 (p = 0.345)	37.50 ± 3.44
Percentage rates of fertilization, 2-cell, 8-cell, and blastocyst formation of control and PCOS mice oocytes cultured in maturation medium, supplemented with 0, 1, 50, and 100 μg/ml NSE. NES: Nigella sativa extract; PCOS: Polycystic ovary syndrome. Data are presented as Mean ± SEM. P-value are presented between two groups (Control versus PCOS) by student's *t* test								

**Table 5:** The effect of different concentrations of NSE on embryonic development in the control and PCOS mice oocytes during in vitro maturation


**Groups**	**Fertilization (%)**		**2-cell (%)**		**8-cell (%)**		**Blastocyst (%)**	
	**Control**	**PCOS**	**Control**	**PCOS**	**Control**	**PCOS**	**Control**	**PCOS**
**Control (0)**	78.50 ± 2.45	68.50 ± 2.34	66.83 ± 2.84	55.16 ± 3.57	49.00 ± 4.11	40.67 ± 4.12	42.50 ± 3.16	30.83 ± 2.54
**NES 1 μg/ml**	87.50 ± 2.67 (p = 0.112)	75.16 ± 2.18 (p = 0.225)	73.17 ± 3.52 (p = 0.276)	69.83 ± 2.77 (p = 0.167)	51.66 ± 3.47 (p = 0.368)	45.00 ± 4.10 (p = 0.651)	46.33 ± 2.86 (p = 0.425)	37.00 ± 3.52 (p = 0.327)
**NES 50 μg/ml**	88.83 ± 1.85 (p = 0.145)	82.17 ± 1.9 (p = 0.027)	81.50 ± 2.86 (p = 0.025)	77.50 ± 2.41 (p = 0.015)	65.00 ± 3.01 (p = 0.033)	63.33 ± 3.86 (p = 0.017)	57.83 ± 2.70 (p = 0.037)	50.33 ± 2.94 (p = 0.008)
**NES 100 μg/ml**	76.17 ± 3.10 (p = 0.625)	71.17 ± 2.98 (p = 0.341)	69.00 ± 3.52 (p = 0.725)	59.50 ± 3.22 (p = 0.635)	48.00 ± 3.61 (p = 0.976)	44.67 ± 3.45 (p = 0.725)	42.50 ± 3.56 (p = 0.951)	37.50 ± 3.44 (p = 0.542)
Percentage rates of fertilization, 2-cell, 8-cell, and blastocyst formation of control and PCOS mice oocytes cultured in maturation medium, supplemented with 0, 1, 50, and 100 μg/ml NSE. NES: Nigella sativa extract; PCOS: Polycystic ovary syndrome. Data are presented as Mean ± SEM. P: P-value between each concentration group of NSE and control (0 μg/ml of NSE) by one-way ANOVA								

## 4. Discussion

The present research showed that maturation, developmental competency, epigenetic alteration, and oxidative statues were significantly different between the control and PCOS mice oocytes. Moreover, these results showed that 50 µg/ml NS NSE in culture medium was associated with a significant increase in *Dnmt1*, *Hdac1, Cdk1, Mapk,* and* Gpx1 *genes and a significant decrease of *Cox2* gene expression of mature oocytes. Moreover, we found that both 1 and 50 µg/ml NSE in the PCOS and control mice had lower levels of ROS and higher levels of GSH than those in other groups during oocyte maturation. Since *cdk1* and *Mapk* are two key genes in oocyte maturation (22), in addition to the fact that *Dnmt1* and *Hdac1* are two essential genes for normal epigenetic modification and development (11), the difference in the maturation fertilization and embryonic development outcome may be partly induced by the expression alterations of genes in the oocytes of the PCOS and control groups.

According to a relevant report by Salarinia and colleagues, treatment of ovary cells with 12.5-200 μg/mL NSE had no significant effect on the viability of these cells (23). Based on work of research, we tried to choose the most appropriate NSE concentration in the culture medium of oocyte maturation. To evaluate oocyte maturation, normal and induced PCOS mice oocytes were exposed to different NSE concentrations added to the IVM medium. Our results showed that NSE could improve the rate of maturation, as well as those of fertilization and blastocyst formation in in-vitro-matured oocytes in both control and PCOS mice. In our pilot experiment, the IVM medium was supplemented with 0-100 µg/ml NSE. Then, the dose-dependent effects of NSE concentrations from 0 to 100 µg/ml (0, 1, 10, 50, 70, and 100 µg/ml) were assessed. Maturation and normal morphology rates were progressively improved as the concentration of NSE increased up to 50 µg/ml, but then the rates decreased to concentrations of 70 and 100 µg/ml NSE. Thus, 0, 1, 50, and 100 µg/ml NSE concentrations were added to the culture medium to evaluate oxidative stress, epigenetic alteration, and embryonic development. A comparison of oocyte maturation for different doses of NSE showed that lower doses could enhance the maturation rate and, therefore, a high concentration of NSE may be linked to abnormal morphology rate. Besides, NSE acts in a dose-dependent manner, which is associated with the pathological condition of the body.

The protective activity of NS oil in reproductive organs is evaluated by Al-Seeni et al., who observed NS oil induced regeneration of spermatogenesis in seminiferous tubules in tartrazine treated rats (24). Also, testicular toxicity after colchicine exposure in rats was prevented by NS oil (25). Another study Besides, the protective effect of NS against cyclophosphamide induced toxicity on the testicular and acrosomal function of spermatozoa in mice (26). Pharmacological studies demonstrated that NS could be involved in the induction of antioxidant mechanisms, thus, preventing the oxidative stress of adverse conditions (8). It was previously shown that NSE of NS increased fertility potential as well as LH and testosterone concentration in male rats, all accompanied by the induction of gonadotropin activity (16). Likewise, the prophylactic effects of NS against cyclophosphamide-induced female mice were suggested by the increase in primary and secondary follicles in ovarian volume (27). Thus, NS reduces the adverse effects of *in-vivo* pathological conditions as well as in-vitro adverse manipulations.

In vitro oocyte maturation was conducted by nuclear and cytoplasmic maturation. *MPF* and *MAPK* play an important role in oocyte maturation because they regulate cell cycle progression during both mitosis and meiosis. These kinas activities are essential for proper fertilization and preimplantation embryo development (28). We did not measure kinase activity levels; instead, we evaluated the *Cdk1* as catalytic subunit, *cyclin B* as a regulatory subunit of *MPF* and *Mapk *gene expression using real-time PCR method. We showed that although NSE had a significant effect on nuclear oocyte maturation by inducing the overexpression of *Cdk1* and *Mapk *genes, it did not have any effect on *cyclin B *gene expression. A previous report in bovine oocyte maturation showed that *Cdk1* is upregulated in IVM medium supplemented with resveratrol as an antioxidant (19). Moreover, the addition of L-carnitin in IVM medium had a significant effect on the overexpression of *Cdk1* gene in mice oocytes (29).

Synthesis and accumulation of GSH content as a non-enzymatic antioxidant in the ooplasm play a key role in cytoplasmic maturation and regulating sperm decondensation. Moreover, GSH is necessary for the replacement of protamine by histone protein in sperm head chromatin after sperm penetration. Thus, oocyte-developing competence and fertilization rate in cytoplasmic level are associated with the intracytoplasmic content of GSH (3). In the present study, supplementing IVM medium with 50 µg/ml NSE increased fertilization rate in oocytes. This finding may be partly explained by the upregulation of GSH level and its insufficient expression which can lead to fertilization failure. Other recent studies also showed that NSE could induce GSH in streptozotocin-induced diabetic male Wistar rats and also improve the adverse conditions resulting from tartrazine administration (24, 30). Due to its nature, NSE can increase the activity of CAT and GSH levels against cisplatin-induced renal toxicity and oxidative stress in Wistar rats (31).

Oocytes and embryos are equipped with both enzymatic and non-enzymatic antioxidants that are crucial for the acquisition of full developmental competency and avoiding or slowing the initiation of apoptosis (32). In the present study, exposure to NSE with 50 µg/ml in the IVM medium was found to be associated with an induction in *Gpx1 *gene expression, an enzymatic antioxidant in the oocytes indicating an improved function of the oocyte antioxidant defense system. These results are consistent with Busari et al., who illustrated that NSE affected the activity of enzymes constituting cell antioxidative system (31). Supplementation of cyclophosphamide-induced rats with NS oil regulated the assayed antioxidants, representative of its ability to reestablish antioxidative homeostasis (33). It was reported that NSE could induce the activity of several antioxidant enzymes such as CAT, GPx, and GST as well as scavenge free radicals. Other studies have shown that TQ, an active component of NS, can overcome the oxidative stress induced during diethylnitrosamine metabolism by upregulation of GST, GPx, and CAT genes in hepatic cells (6).

Another study clearly confirmed that administering TQ resulted in a significant progression of normal follicular development of PCOS rats. This result was obtained from suppressing NF-ĸB signaling which inhibits *Cox2* expression (34). It was suggested that DHEA cab induces the overexpression of *Cox2* in PCOS mice model (35). The overexpression of *Cox2* is positively correlated with ROS production which coincided with the change of cytoplasmic development and apoptosis induction (36). The data presented here show that NSE reduced *Cox2 *expression and avoided ooplasmic ROS production induced by both DHEA and manipulation in culture condition in both control and PCOS mice oocytes. Additionally, in agreement with our results, Arif et al. found that exposure of granulose cell line with TQ could inhibit ROS production and the overexpression of *Cox2* gene (34). This unique property of NSE, as an herbal antioxidant to inhibit the negative effect of ROS production, highlights its pivotal role in antioxidant activity besides its effect on free radical scavenging.

In our previous study, mice treated with DHEA showed a significant increase in the state of oxidative stress and epigenetic alteration compared to a control after three weeks. The increase of ROS generation, linked to the downregulation of *Hdac1* gene, resulted in an aberrant epigenetic pattern in DHEA-treated oocytes (15). Inhibition of histone deacetylation mediated by the Hdac1 enzyme during meiosis may be linked to the high incidence of aneuploidy and embryo abnormality (37). The results of the present study showed that treatment with 50 µg/ml NSE in culture medium increased epigenetic enzymes including *Dnmt1* and *hdac1*, compared with the untreated oocytes. It is notable that epigenetic enzymes regulate the methylation of 5-methyl cytosine and histone proteins leading to methylated oocyte genome, and finally the condensation of chromosome (11). As we know, manipulative procedures such as IVM resulting in damages related to nuclear-cytoplasmic interactions may alter the correct performance of these epigenetic processes and lead to abnormal embryo development (38). Therefore, prevention of adverse constituents such as ROS production via modifying culture medium might provide further insight into the epigenetic modifications that occur during oocyte maturation.

## 5. Conclusion

Maturation, developmental competency, oxidative stress, and epigenetic modification were significantly different between mice oocytes in the control and PCOS roups. The present study showed that NSE improves specific development competence. Differences in the features of oocyte maturation and developmental competence were reduced for mice both in the control and PCOS when NSE was supplemented at the dose of 50 µg/ml concentration during IVM. Our results showed that the beneficial effects of NSE are dose-dependent and increase at the concentration of 50 µg/ml during oocyte maturation. Further studies are required to explore NSE effects on mice in control and PCOS groups.

##  Conflict of Interest

None to declare.
